# Proteomic Shifts in Embryonic Stem Cells with Gene Dose Modifications Suggest the Presence of Balancer Proteins in Protein Regulatory Networks

**DOI:** 10.1371/journal.pone.0001218

**Published:** 2007-11-28

**Authors:** Lei Mao, Claus Zabel, Marion Herrmann, Tobias Nolden, Florian Mertes, Laetitia Magnol, Caroline Chabert, Daniela Hartl, Yann Herault, Jean Maurice Delabar, Thomas Manke, Heinz Himmelbauer, Joachim Klose

**Affiliations:** 1 Institute for Human Genetics, Charité-University Medicine Berlin, Germany; 2 Max Planck Institute for Molecular Genetics, Berlin, Germany; 3 Institut de Transgénose, IEM, UMR6218, CNRS Uni Orléans, Orléans, France; 4 EA 3508, Université Paris Diderot-Paris 7, Paris, France; Children's Hospital Boston, United States of America

## Abstract

Large numbers of protein expression changes are usually observed in mouse models for neurodegenerative diseases, even when only a single gene was mutated in each case. To study the effect of gene dose alterations on the cellular proteome, we carried out a proteomic investigation on murine embryonic stem cells that either overexpressed individual genes or displayed aneuploidy over a genomic region encompassing 14 genes. The number of variant proteins detected per cell line ranged between 70 and 110, and did not correlate with the number of modified genes. In cell lines with single gene mutations, up and down-regulated proteins were always in balance in comparison to parental cell lines regarding number as well as concentration of differentially expressed proteins. In contrast, dose alteration of 14 genes resulted in an unequal number of up and down-regulated proteins, though the balance was kept at the level of protein concentration. We propose that the observed protein changes might partially be explained by a proteomic network response. Hence, we hypothesize the existence of a class of “balancer” proteins within the proteomic network, defined as proteins that buffer or cushion a system, and thus oppose multiple system disturbances. Through database queries and resilience analysis of the protein interaction network, we found that potential balancer proteins are of high cellular abundance, possess a low number of direct interaction partners, and show great allelic variation. Moreover, balancer proteins contribute more heavily to the network entropy, and thus are of high importance in terms of system resilience. We propose that the “elasticity” of the proteomic regulatory network mediated by balancer proteins may compensate for changes that occur under diseased conditions.

## Introduction

Investigations of etiology and pathogenesis of human diseases are frequently performed using suitable animals as a model system. Most commonly mice are employed where a gene of particular interest is knocked out, mutated or overexpressed. When the effect caused by genome modification is subsequently studied in these mice at the molecular level, usually a large number of changes are observed on the mRNA and protein levels, in spite of the fact that only a single gene was altered. For example, in protein patterns obtained by two-dimensional gel electrophoresis (2-DE) of brain proteins from a mouse model for Parkinson's disease deficient of the parkin protein [1] and from a transgenic mouse model for Huntington's disease [2], we detected 15 and 40 variant proteins, respectively [3], [4]. Using more sensitive protein detection methods, such as the differential in-gel electrophoresis (DIGE) technique and analyzing two different brain regions at two different age stages, 87 quantitatively variant proteins were detected in the parkin knock-out mouse [5]. In investigations of a transgenic mouse model for Alzheimer's disease that overexpressed mutated human amyloid precursor protein (*App*) [6] using our large-gel 2-DE [7], [8] and DIGE technique, we detected more than one hundred variant proteins (Hartl D. et. al., unpublished results). On the mRNA level, Miller and colleagues observed over 600 changes in a single gene modified Parkinson disease mouse model [9]. Similar results were also obtained in other single gene knock-out mouse models [10].

Apparently, the molecular response to a single gene mutation is of considerable complexity, and certainly much more complex than detectable using current experimental approaches. We have previously compared the protein changes detected in mouse models for different neurodegenerative diseases and, in addition, mouse models of non-neurodegenerative disorders [11]. We found that up to 36% of variant proteins were shared among these different disease models and hypothesized that these protein alterations were not disease-specific. Unexpectedly, when we compared wild-type mice of different inbred strains, we found that most of these putative disease-unspecific protein alterations also occurred as polymorphisms that distinguished strains of mice. This suggested that some, if not most of the protein changes observed when investigating disease models might not be genuinely informative regarding etiology or pathogenesis of the disease under consideration.

To investigate the significance of protein changes under disease conditions, we have chosen a more systematic and simplified approach by using mouse embryonic stem (ES) cells with highly defined modifications in a controlled environment. Six mutant cell lines were investigated. All of them contained gene modifications relevant to neurodegenerative diseases. Four cell lines contained one single overexpressed gene, i.e. *App* (a cell surface receptor), *Snca* with changes relevant to Alzheimer's and Parkinson's disease, respectively [9], [12] and *Dyrk1a* (a nuclear kinase) as well as *Dopey2* (a leucine zipper-like protein) both relevant to Down syndrome [13], [14]. In two other cell lines, a segment encompassing 14 genes relevant to Down syndrome was duplicated (trisomic) in one case and deleted (monosomic) in the other [15]. The six mutant cell lines were investigated by 2-DE and altered protein expression was recorded by comparison with the respective parental lines. Many variant proteins showing up or down-regulation were observed. Profound quantitative analysis of protein changes led us to the hypothesis that the cellular proteome is kept quantitatively in balance by a particular class of proteins to which we refer as “balancer proteins”. Accordingly, we assume that when the quantitative arrangement of the proteome is perturbed by gene dosage effects, it will be subjected to a rearrangement in order to achieve a new balance. Thus, the many protein changes observed may reflect the rearrangement of the proteome to protect the cell from deleterious effects of gene dosage mutations.

## Results

**Figure 1:**
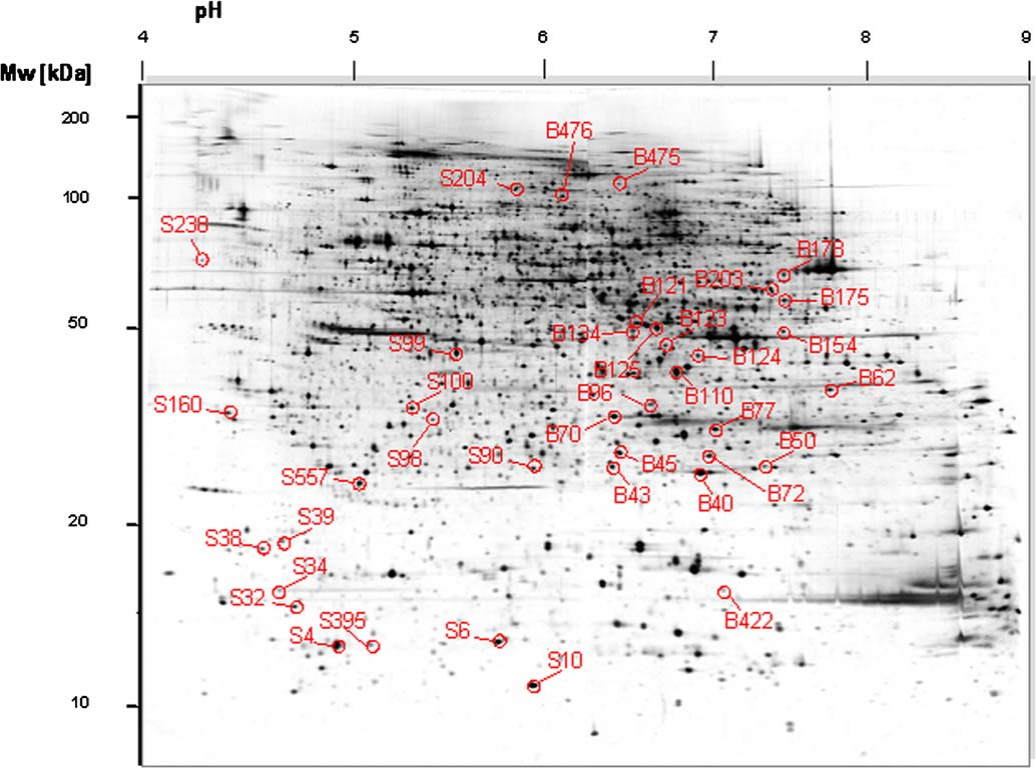
Representative protein expression pattern of mouse embryonic cell lines as revealed by large-gel 2D-electrophoresis. Over 5500 proteins (including protein isoforms) were resolved on a single gel. Highlighted spots correspond to spot ID of candidate balancer proteins detailed in Table 2.

Proteins expressed in ES cells were separated by large-gel 2-DE. On a representative 2-DE pattern of total protein extract from ES cells, a total of 4958 protein spots could be scored visually (Figure 1). Using Delta2D imaging software (see Methods), over 5500 protein spots were detected. Six different transgenic cell lines were investigated in this study. These comprised two cell lines in which one single gene was duplicated (*mES_Dyrk1a_Tris* or *mES_Dopey2_Tris*), and two cell lines in which one gene was overexpressed (5.5 times more than wild-type in *mES_hAPP* or 1.6 times in *mES_Snca*). In two cell lines gene dosage was altered over a chromosomal region that spanned 14 genes on mouse chromosome 17. A hemizygous deletion line was monosomic for the interval (*mES_14_Mono*). The other line contained an engineered duplication of the segment, and thus was trisomic (*mES_14_Tris*). No difference was observed between transgenic and parental cell lines with respect to cellular morphology and growth behavior. The six cell lines were compared to their parental cell lines with regard to their protein expression profiles. The number of proteins that showed significantly increased or decreased expression, when compared to their expression in parental cell lines, was in the range of 70 to 110 variants per cell line (Table 1). In total, 255 distinct variant proteins were observed in the six cell lines (Table S1). The data-adjusted modified t-test SAM (Significance Analysis of Microarrays) was used to calculate that the false discovery rate for obtaining a comparable result was less than 1 %.

**Table 1:** Number of quantitatively variant proteins in six transgenic mouse embryonic stem cell lines.

Quantitative changes	Number of variant proteins in different transgenic cell lines					
	mES_14_Mono*	mES_14_Tris*	mES_Dopey2_Tris	mES_Dyrk1a_Tris	mES_hAPP	mES_Snca
**Up-regulated**	44	44	37	41	46	52
**Down-regulated**	62	26	45	37	47	57
**Total**	106	70	82	78	93	109

* The segment from mouse chromosome 17 includes 14 genes.

**Table 2:** Proteins changed in more than three transgenic ES cell lines (proposed balancer proteins).

Spot ID	Protein Name	Gene Symbol	Behavior
B125	aminolevulinate, delta-, dehydratase	*Alad*	3 ↑, 2↓
S37	albumin	*Alb*	1 ↑, 3↓
B40	ATPase, H+ transporting, lysosomal V1 subunit C1	*Atp6v1c1*	always up
S34	BAT2 domain containing 1	*Bat2d*	always down
B96	carbonic anhydrase 2	*Car2*	1 ↑, 3↓
B476	calcium response factor	*Carf*	2 ↑,2↓
B72	coiled-coil domain containing 25	*Ccdc25*	always up
B178	eukaryotic translation elongation factor 1 alpha 1	*Eef1a1*	1 ↑, 3↓
B70	enolase 1, alpha non-neuron	*Eno1*	always up
S10	fatty acid binding protein 3, muscle and heart	*Fabp3*	2 ↑, 4↓
B110	guanine nucleotide binding protein (G protein), beta polypeptide 2 like 1	*Gnb2l1*	1 ↑, 3↓
S98	golgi autoantigen, golgin subfamily b, macrogolgin 1	*Golgb1*	1 ↑, 4↓
B175	glutamate oxaloacetate transaminase 2, mitochondrial	*Got2*	3 ↑, 1↓
B121	glyoxylate reductase/hydroxypyruvate reductase	*Grhpr*	3 ↑, 2↓
S160	histone cell cycle regulation defective interacting protein 5	*Nfu1*	2 ↑, 2↓
B154	heterogeneous nuclear ribonucleoprotein A2/B1	*Hnrpa2b1*	4 ↑, 1↓
B134	LIM and SH3 protein 1	*Lasp1*	2 ↑, 4↓
B123	mitochondrial ribosomal protein L39	*Mrpl39*	3 ↑, 2↓
S38	nucleophosmin 1	*Npm1*	1 ↑, 3↓
B62	nudix (nucleoside diphosphate linked moiety X)-type motif 16-like 1	*Nudt16l1*	always up
B475	polyribonucleotide nucleotidyltransferase 1	*Pnpt1*	1 ↑, 3↓
S238	pyrophosphatase (inorganic) 1	*Ppa1*	2 ↑, 2↓
B45	PPAR-alpha interacting complex protein 285	*Pric285*	4 ↑, 1↓
S557	proteasome (prosome, macropain) subunit, beta type 6	*Psmb6*	always down
B77	proteasome (prosome, macropain) subunit, beta type 7	*Psmb7*	always up
S90	proteasome (prosome, macropain) 28 subunit, alpha	*Psme1*	1 ↑, 3↓
B43	RAN binding protein 5	*Ranbp5*	always up
S6	S100 calcium binding protein A11 (calgizzarin)	*S100a11*	always up
B203	serine (or cysteine) peptidase inhibitor, clade H, member 1	*Serpinh1*	1 ↑, 3↓
B422	single-stranded DNA binding protein 1	*Ssbp1*	2 ↑, 2↓
B50	transgelin	*Tagln*	1 ↑, 3↓
B124	transaldolase 1	*Taldo1*	3 ↑, 2↓
S32	transcription elongation factor B (SIII), polypeptide 2	*Tceb2*	always down
S204	thimet oligopeptidase 1	*Thop1*	1 ↑, 3↓
S100	tropomyosin 1, alpha	*Tpm1*	1 ↑, 4↓
S395	Thioredoxin-like 2	*Txn1*	2 ↑, 2↓
S4	Thioredoxin 1	*Txn1*	2 ↑, 2↓
S99	Thioredoxin-related protein	*Txnl1*	2 ↑, 2↓

**Figure 2:**
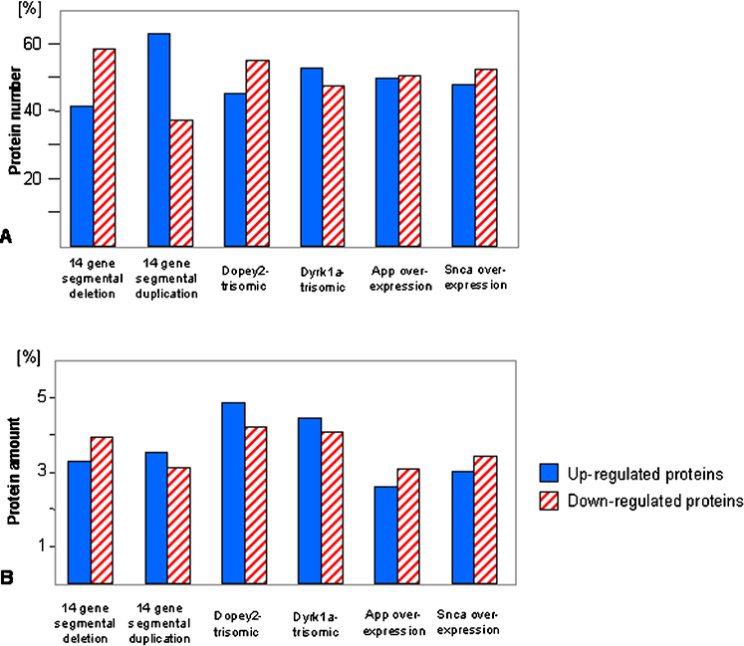
Proteins that showed altered expression in transgenic ES cell lines. (A) Number of altered proteins in each transgenic cell line, expressed as percentile of total number of altered proteins. (B) Amount of proteins that underwent altered expression in each cell line, represented as percent of total spot volume that was up or down-regulated in transgenic cell lines. Dose alteration of 14 genes could no longer be balanced by an equivalent number of variant proteins. However, a balance remained at the level of protein concentration.

In the four cell lines that overexpressed a single gene, 40 to 50 proteins were up-regulated. This was always accompanied by a similar number of down-regulated proteins. A quite different situation was found for the two cell lines with the dosage alteration in 14 genes: If duplicated, 60% of proteins were up-regulated and 40% were down-regulated (40%). In case of deletion, a similar imbalance was found, but in the opposite direction, i.e. about 60% of the variant proteins showed decreased expression, while only about 40% were over-expressed (Figure 2A). The observations described above were based on the number of proteins showing altered expression profiles in the transgenic cell lines. In the next step, we investigated the total protein amount showing altered expression within each cell line by determining relative protein concentrations (protein spot volumes) across all altered proteins. This resulted in a balanced picture, i.e. no significant difference could be detected in the protein amount undergoing up and down-regulation (Figure 2B). Most importantly, this was even true for the two cell lines with 14 genes altered, which showed a drastic imbalance in the number of proteins that underwent up or down-regulation (see above).

**Figure 3:**
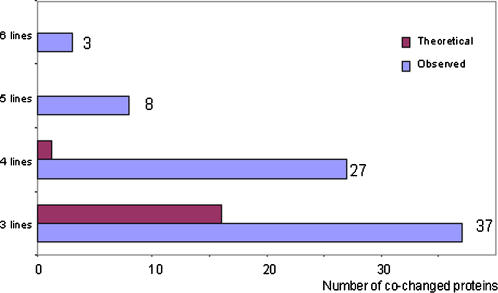
Comparison of observed number of co-changed proteins against a theoretical calculation of co-changed proteins across six different transgenic cell lines. It was assumed that a total of 800 protein spots were investigated, among which 10% of the proteins change in their expression profile. This comparison shows that the occurrence of the same protein alteration in more than three cell lines is unlikely to be coincidental.

When we compared proteins that showed quantitative changes among the six cell lines, we found that many of these proteins were altered in several cell lines. Specifically, 38 proteins showed changes in more than three cell lines. Among them, the expression of three proteins changed in all six cell lines, eight proteins changed in five, while 27 proteins changed in four of six different transgenic ES cell lines. In contrast, 114 proteins were altered only in one cell line. In order to test to which extent changes of expression in the same proteins may occur by chance in multiple cell lines independently, the numbers of observed co-changed proteins in different numbers of cell lines were compared to theoretical numbers of co-changed proteins, assuming that a total of 800 protein spots were investigated, among which 10% were differentially expressed in transgenic and control cell lines (Figure 3). Our calculation showed that the occurrence of the same protein alteration in more than three cell lines was unlikely to be coincidental (p<0.001).

An interesting observation was made when we considered proteins that were only altered in both *mES_14_Mono* and *mES_14_Tris*: Two thirds of them showed the same change tendency, i.e., either up-regulated in both cell lines, or down-regulated in both cell lines, despite opposite gene dose alteration (trisomy versus monosomy). This suggested that many changes could be unrelated with respect to the gene(s) that caused the dosage imbalance. Hence, we hypothesize that the proteins showing changes in several cell lines (38 proteins, see above) represent a particular class of proteins, which we propose to call “balancer” proteins (Table 2). Different from that, proteins that were altered only in a single cell line are called here “cell line-specific proteins” to denote protein alterations specific to a cell line characterized by a distinct genetic alteration (114 proteins, see above).

Among the candidate balancer proteins, seven of them were always increased in their expression in our experiment (Table 2). They are: *Atp6v1c1*, *Ccdc25*, *Eno1*, *Nudt16l1*, *Psmb7*, *Ranbp5* and *S100a11*. On the other hand, three balancer proteins (*Bat2d*, *Psmb6* and *Tceb2*) were consistently down-regulated in their expression. One protein (*Psme1*) was down-regulated in three cell lines with transgene overexpression (*mES_14_Tris*, *mES_Dyrk1a_Tris* and *mES_Snca*), while it was up-regulated in *mES_14_Mono*. To determine whether putative balancer or cell line-specific proteins might be direct interaction partners of genes mutated in the six cell lines, we queried all mutated genes, balancer proteins and cell line-specific proteins in the KEGG pathway database. No overlapping KEGG pathway entries were detected between balancers and mutated genes. On the contrary, four KEGG pathway terms of cell line-specific protein overlapped with that of mutated genes in our six ES cell lines. These included methionine metabolism, selenoamino acid metabolism, ABC transporters and purine metabolism. Similar conclusions could be drawn from Biocarta pathway database queries.

**Figure 4:**
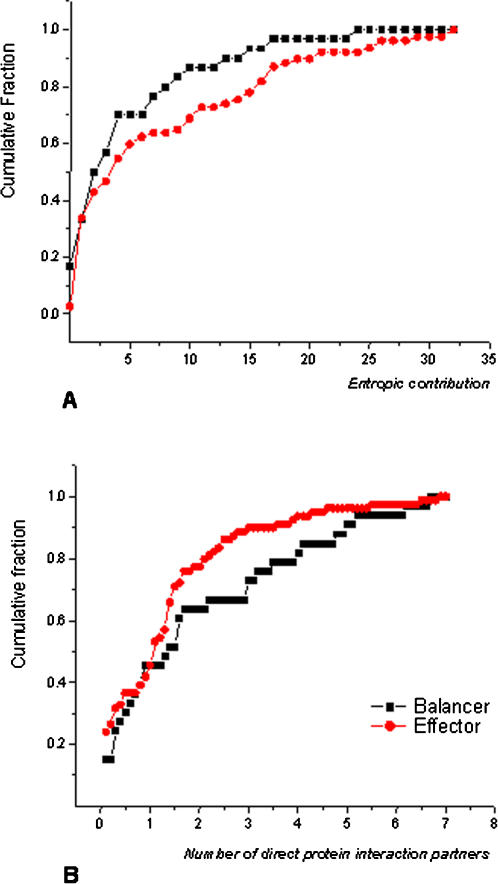
Cumulative fraction plots of “balancer”-“cell line-specific protein” comparison. (A) Entropic contribution.; (B) Number of direct protein interaction partners. Compared to cell line-specific proteins, balancers possess significantly higher values of entropic contribution and a low number of direct interaction partners.

In order to investigate whether balancers and cell line-specific proteins might represent two different classes of proteins with certain biochemical and biophysical properties, we compared these two sets according to different parameters. The spectrum of biochemical and biophysical criteria selected for characterization included molecular weight, isoelectric point, predicted protein instability, aliphatic index, hydrophobicity, cellular abundance, polymorphisms (i.e. allelic diversity) and number of direct protein interaction partners. As summarized in Table 3, balancers and cell line-specific proteins showed no perceivable difference in their molecular weights and isoelectric points, neither in their instability, nor regarding aliphatic index or hydrophobicity. However, balancer proteins were significantly more abundant in the cell (p = 0.008). Furthermore, cell line-specific proteins were found to have twice as many interaction partners as balancer proteins (p = 0.004) (Figure 4B). We then queried the Mouse Genome Informatics Database (www.informatics.jax.org) for the occurrence of single nucleotide polymorphisms (SNPs) in balancer and cell line-specific proteins as a measure of their allelic diversity. Interestingly, the potential balancers had significantly more non-synonymous SNPs in coding regions than potential cell line-specific proteins (Table 3), while no significant difference could be established for other SNP evaluations (total number of SNPs, proportion of synonymous SNPs in the coding regions and the frequency of SNPs in the 5′-UTR, 3′-UTR, introns and sequences flanking upstream and downstream of a locus).

**Table 3:** Comparison of protein properties of balancer and cell line-specific proteins.

Protein properties	Balancers	Cell line-specific proteins	p-value
**Molecular weight** (kD)	47.2±42.5	48.3±47.3	0.906
**Isoelectric point**	6.52±1.68	6.98±1.71	0.14
**Instability**	41.3±12.8	41.8±11.2	0.81
**Aliphatic index**	79.7±14.2	77.7±16.8	0.51
**Gravy score**	−0.449±0.337	−0.444±0.357	0.94
**Cellular abundance** (% volume of protein spot)	**0.158±0.169**	**0.118±0.116**	**0.0082**
**No. of interaction partners** (3)	**4.9**	**7.8**	**0.0048**
**Total No. of SNPs per locus**	29.5±40.1	30.6±58.7	0.46
**No. of upstream SNPs ** (1)	2.7±4.7	1.9±4.9	0.53
**No. of SNPs in 5′-UTR**	1.0±2.3	1.9±4.9	0.76
**No. of SNPs in introns**	18.8±31.3	20.5±48.1	0.43
**No. of synonymous SNPs in ORF**	1.3±3.1	1.4±0.5	0.12
**No. of nonsynonymous SNPs in ORF**	**2.8±1.83**	**0.7±1.8**	**0.026**
**No. of SNPs in 3′-UTR**	2.1±4.6	1.1±3.2	0.0855
**No. of SNPs downstream ** (2)	2.6±5.4	3.2±5.3	0.89
**Entropic contribution** (3)	**1.51**	**0.97**	**0.02**

Values in bold indicate significant difference between balancer and cell line-specific proteins

(1) Interval up to a position 2000 bp upstream of the transcription start site

(2) Interval from polyadenylation site to a position 2000 bp downstream

(3) Standard errors not shown since the distributions are tend to be screwed.

To assign functional categories, a Gene Ontology (GO) term enrichment analysis was performed. Tables 4 and 5 give a summary of GO-terms that occurred more frequently in balancers or cell line-specific proteins, respectively, based on human GOA database searches (see Methods for details). Eight GO-terms were specifically over-represented in balancer candidates. They comprise protein degradation, disulfide modification and electron carrier processes. In contrast, over 33 GO-terms were enriched in cell line-specific proteins. Notably, a large part of them were involved in mRNA processing and related functions. These two GO-term sets overlap by participating in protein chaperoning of catabolism processes.

**Figure 5:**
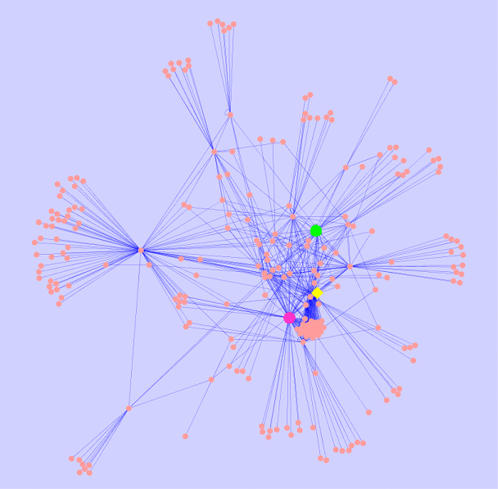
A protein-protein interaction subgraph showing the proteasome subunits, where nodes denote proteins and the edges describe protein-protein interaction. Two local hub proteins of this subgraph (Psma2 and Psma3) belong to cell line-specific proteins, while a candidate balancer protein (Psmb6) represents a connection between these two modules (see discussion for details). This supports our assumption that balancer proteins could be connective hubs between different modules. Protein marked in green: Psma2; yellow: Psmb6; magenta: Psma3.

Next, we undertook an analysis of protein-protein interactions that balancer and cell line-specific proteins participate in, chiefly based on the Human Reference Protein Database (see methods for details). The protein-protein interaction graph constructed from our ES cell data comprised 2677 nodes (distinct proteins, indicated by gene symbol). This interaction graph shared the common feature of scale-free geometry with other protein interaction networks, such as that of *E. coli* or *Saccharomyces cerevisiae* [16], [17]. Among all protein nodes, 2565 (96%) of them could be linked to a giant network component with heterogeneous degree distribution. The remaining 112 proteins formed 41 isolated components, with the number of nodes varying from one to twelve. Figure 5 shows a subset of the protein-protein interaction network centered around the proteasome subunits. In the entropy analysis of the network, we focused on the giant network component, since the network entropy is only defined for the strongly connected components of the network. All 38 balancer proteins belonged to the giant network component, as well as 79 out of 114 cell line-specific proteins.

As network entropy is a measure of system homeostasis, we may expect high-ranking proteins to be affected more frequently as the cell responds to various stimuli. Through a direct comparison of balancers to cell line-specific proteins using their entropic contribution, we found that balancers, on average, possess significantly higher values of entropic contribution than cell line-specific proteins (p = 0.02, Wilcoxon rank test, Figure 4). Alternatively, we ask to what extent the entropic measurement can distinguish between cell line-specific proteins and balancers within the background of all proteins in the giant component. To this end, we took the same number of top-ranking proteins based on their entropic contribution and studied their overlap with our 38 balancers or 79 cell line-specific proteins, respectively. Assuming a hypergeometric distribution over a total of 2526 proteins, this corresponds to p = 0.018 and p = 0.094 for balancers and cell line-specific proteins, respectively. This illustrates that the entropic ranking of proteins selects balancers preferentially, thus it validates our previous observation that proteins with high contribution to network entropy are enriched in the set of balancer proteins.

## Discussion

We investigated the effect of gene dosage alterations on the proteome of mouse embryonic stem (ES) cells. Using our large-gel 2-DE, extraordinary in its high resolution and reproducibility [7], total protein extracts from six different ES cell lines were analyzed. In four of them, one single gene was overexpressed either by gene duplication (*Dyrk1a*, *Dopey2*) or by conventional stable gene transfection (*App*, *Snca*). In two other cell lines, the dosage of a whole set of 14 genes was altered so that the segment was either duplicated (trisomic) or deleted (monosomic). According to our observations, dose alteration of a single gene led to quantitative changes in a large number of proteins. Surprisingly however, altering the dosage of 14 genes instead of one gene did not increase the number of altered proteins accordingly. In effect, the frequency of protein variations induced by one or 14 altered gene dosages was in a similar range. Hence, we propose that the protein changes observed might not completely reflect reactions of proteins functionally linked with the genes whose dosage was altered. Rather, these changes may at least be partially explained as a global response of the cellular proteome to the gene dosage defect.

Considering the protein changes observed in our ES cell lines in more detail, we found that in all cases where a single gene was overexpressed, the number of proteins which were up-regulated was always in equilibrium with the number of down-regulated proteins (Figure 2A). Moreover, when we measured up and down-regulation of proteins in terms of protein amount instead of number of proteins, a balance in up and down-regulation was also observed. The situation was different in the two cell lines carrying alterations in 14 genes. Here, the number of proteins up or down-regulated was no longer in equilibrium: In *mES_14_Tris*, about 60% of the altered proteins were up-regulated, whereas about 40% of the proteins were down-regulated. The changes in the *mES_14_Mono* showed the same ratio, but in reversed direction (ca. 60% down, 40% up). However, regarding the protein variations at the level of protein amount, a balance reoccurred even in cell lines with 14 genes altered (Figure 2B).

We therefore hypothesize the existence of a proteome-wide acting regulatory mechanism that leads to a compensation of an imbalance in the quantitative arrangement of the cellular proteome. Within the proteome of a cell, the relative concentration of each particular protein should be precisely arranged and well balanced. In consequence, aberrant quantitative changes, even in a single protein, may alter the relative concentration of many other proteins, thereby disturbing the overall proteomic balance. In this situation, the first response of the cell could be towards restoring the balance in the cellular proteome in order to maintain normal cellular operations. As a result, below certain thresholds, a rebuild of system homeostasis by quantitative rearrangement of the proteome may be achieved.

Several considerations that originate from theoretical biology and experimental model systems are in line with our hypothesis outlined above: The theory of protein minimization [18] states that all protein levels within a cell are maintained at the minimum level compatible with function, while metabolic pathway fluxes are maintained at the maximum. This is explained as a consequence of an increasing number of proteins occurring in the course of evolution, e.g. by gene duplication, that needed to be accommodated in the cells. Since the resources of a cell (such as space, energy, metabolites e.g. amino acids and unbound water to allow diffusion) did not increase accordingly, the occurrence of new proteins in evolution was always accompanied by a concentration reduction of proteins that already present. In order to keep cellular functions intact in spite of protein concentration reduction, the functional efficiency of the already established proteins (e.g. the specific activity of enzymes) had to increase. Another theory, the excluded volume theory established by A.P. Minton [19], [20] deals with the high degree of macromolecular crowding in cells. If a protein is overexpressed in a cell, movement of this and surrounding proteins becomes restricted due to excluded volume. Thus the distance between protein molecules becomes smaller than the diameter of moving protein molecules. Proteins react to this situation with conformational changes and tend to aggregate and to lose their function. Apparently, active or passive regulatory mechanisms exist that keep the cellular protein concentrations within a physiologically buffered range.

When the relative cellular proteome composition is disturbed, probably not all proteins are changed in their quantitative occurrence to the same extent. Regarding our hypothesis, we suggest the existence of a special class of proteins that are particularly effective in such rebalancing approaches. This led us to propose discrimination between balancer and cell line-specific proteins. We hypothesize that balancers are proteins that buffer or cushion a cellular system by common properties, i.e., properties not necessarily related to their specific functions. Accordingly, the same proteins may change when different system disturbances have caused protein imbalance. In line with these definitions, we found no considerable overlapping functions between balancer proteins and the transgenes. In contrast, the expression alterations of cell line-specific proteins could more likely have been directly induced by gene dosage modifications. This finding further supports the notion that the changes of balancer proteins represent more peripheral cellular affairs.

To find out whether balancer proteins might have further distinct properties, we analyzed them using multiple categories outlined in tables 3, 4 and 5. We found that potential balancers seem to be of high cellular abundance. This is plausible as very low abundance proteins (e.g. regulatory proteins, transcription factors and receptors) are possibly present only in a few copies per cell and thus have no real buffer capacity to compensate imbalance at the proteomic scale. In retrospect, it is known that all proteins visible on 2-DE patterns are relatively abundant [21]. Still, even under these preconditions, balancer proteins seem to be more abundant than cell line-specific proteins. Moreover, potential balancers turned out to be more polymorphic in their coding regions than cell line-specific proteins. Protein polymorphisms indicate proteins which became less constrained in the course of evolution [22]. As a consequence, proteins bearing a higher degree of polymorphisms (including balancers) may tend to be more flexible in quantitative changes, whereas cell line-specific proteins may require a stronger connection between expression level and function.

Another trait of our candidates for balancer proteins was found by screening a protein-protein interaction database available online (HPRD). Here, balancers possessed less direct interaction partners than cell line-specific proteins. Interestingly, in the protein interaction network published by Stelzl et al. for human proteins [23], disease-related proteins annotated in the *Online Mendelian Inheritance in Man* database (OMIM) were located almost exclusively in the area of low connective level. This correlation may indicate a particular role of balancer proteins in diseases conditions, but at the same time raises our suspicion that balancer proteins are more likely to be identified as disease-associated proteins partially due to their frequent and reproducible alterations.

Towards understanding how balancer proteins in their functional properties may impart elasticity to the proteomic system, we queried what kind of shared functional categories these proteins may possess (biological process and molecular function GO terms). Compared to the candidate balancers, cell line-specific proteins were associated with a much broader spectrum of GO-categories (Table 4 and 5). In addition, cell line-specific proteins but not balancers were highly involved in mRNA-related processes. This is in line with the fact that these processes are tightly regulated. Proteins involved therein are thus prone to concentration alteration, a property incompatible with a role as balancers. Moreover, our set of putative balancer proteins was enriched in stress and metabolic proteins compared to the remaining proteins altered. The physiological activity of a significant subset of cellular proteins is modified by the redox state of regulatory thiol groups. The cellular redox homeostasis depends on the balance between oxidation of thiols through oxygen and reactive oxygen species and reduction by thiol-disulfide transfer reactions. In this respect, it would make sense that potential balancer proteins are enriched in GO categories implicated in disulfide oxidoreductase and thiol disulfide exchange.

**Table 4:** GO-terms enriched among balancer proteins detected in the proteome of mouse ES cells.

GO-ID	Count§	Total#	p-Value	GO-term	Category*
GO:0009056	7	877	0.002	catabolism	BP
GO:0006091	7	1028	0.003	generation of precursor metabolites and energy	BP
GO:0030508	2	5	0.001	thiol-disulfide exchange intermediate activity	MF
GO:0015035	3	73	0.003	protein disulfide oxidoreductase activity	MF
GO:0016836	3	81	0.003	hydro-lyase activity	MF
GO:0016835	3	92	0.004	carbon-oxygen lyase activity	MF
GO:0015036	3	96	0.004	disulfide oxidoreductase activity	MF
GO:0009055	4	289	0.007	electron carrier activity	MF

§ Number of balancer proteins bearing this GO-term.

# Total number of proteins in the human GOA database annotated with this GO-term.

* BP: biological process; MF: Molecular function.

**Table 5:** Enriched GO-terms among cell line-specific proteins detected in the proteome of mouse embryonic stem cells.

GO-ID	Count§	Total#	p-Value	GO-term	Category*
GO:0043170	41	7475	7.85E-07	macromolecule metabolism	BP
GO:0006396	11	503	3.49E-06	RNA processing	BP
GO:0044238	53	11859	3.83E-06	primary metabolism	BP
GO:0016070	12	662	4.71E-06	RNA metabolism	BP
GO:0008152	57	13425	4.76E-06	metabolism	BP
GO:0008614	2	2	1.72E-04	pyridoxine metabolism	BP
GO:0008615	2	2	1.72E-04	pyridoxine biosynthesis	BP
GO:0042816	2	2	1.72E-04	vitamin B6 metabolism	BP
GO:0042819	2	2	1.72E-04	vitamin B6 biosynthesis	BP
GO:0043283	24	4377	0.001	biopolymer metabolism	BP
GO:0006139	27	5422	0.002	nucleobase, nucleoside, nucleotide and nucleic acid metabolism	BP
GO:0016071	6	292	0.002	mRNA metabolism	BP
GO:0006397	5	243	0.006	mRNA processing	BP
GO:0006511	5	248	0.006	ubiquitin-dependent protein catabolism	BP
GO:0019941	5	248	0.006	modification-dependent protein catabolism	BP
GO:0043632	5	248	0.006	modification-dependent macromolecule catabolism	BP
GO:0044260	25	5232	0.007	cellular macromolecule metabolism	BP
GO:0019538	26	5543	0.007	protein metabolism	BP
GO:0000375	4	148	0.007	RNA splicing, via transesterification reactions	BP
GO:0000377	4	148	0.007	RNA splicing, via transesterification	BP
GO:0000398	4	148	0.007	nuclear mRNA splicing, via spliceosome	BP
GO:0006564	2	16	0.009	L-serine biosynthesis	BP
GO:0030530	2	16	0.009	heterogeneous nuclear ribonucleoprotein	BP
GO:0044257	5	288	0.009	cellular protein catabolism	BP
GO:0051603	5	287	0.009	proteolysis during cellular protein catabolism	BP
GO:0003723	14	930	3.83E-06	RNA binding	MF
GO:0008266	2	2	1.72E-04	poly(U) binding	MF
GO:0016018	2	6	0.002	cyclosporin A binding	MF
GO:0050662	5	193	0.002	coenzyme binding	MF
GO:0000166	21	3851	0.003	nucleotide binding	MF
GO:0048037	5	220	0.004	cofactor binding	MF
GO:0003727	2	12	0.006	single-stranded RNA binding	MF
GO:0008144	2	17	0.010	drug binding	MF

§ Number of cell line-specific proteins bearing this GO-term.

# Total number of proteins in the human GOA database annotated with this GO-term.

* BP: biological process; MF: Molecular function.

One particularly important feature of a living system is its resilience against external and internal changes, which, at the molecular level, amounts to perturbations in network parameters. In an attempt to analyze this robustness of the cellular system, we applied a network analysis, which is motivated by concepts from statistical mechanics and dynamical systems theory. Our approach is based on the assumption that biological processes often operate at steady state, which corresponds to the observed phenotype [24]. It has been shown that changes in network entropy, a fundamental statistical property, are positively correlated with system robustness. In turn, the entropic contribution of a protein describes its impact on network integrity. Removal of nodes with high entropic contribution more often result in lethal phenotypes from yeast and *C. elegans* [25]. Our ranking analysis shows that there is a difference between balancers and cell line-specific proteins: Compared to cell line-specific proteins, balancers possessed a higher entropic contribution. This structural property suggests that balancers might be able to attenuate system disturbance more efficiently. The existence of balancer proteins could therefore be responsible for the elasticity of a cellular system.

For example, a number of proteins representing proteasome subunits showed altered expression in our transgenic cell lines. Five of them belong to balancer candidates, while three other proteasome subunits belong to cell line-specific proteins. Considering the proteasome sub-interaction network in detail (Figure 5), we noticed that *Psma2* and *Psma3*, which are local hubs in the subgraph, both belong to candidate cell line-specific proteins. On the other hand, *Psmb6* is a candidate balancer protein connecting between two different nodes of a higher order. This example supports our assumption that balancer proteins could be connective hubs between different modules. Such “bridges” are probably heavily utilized during balancing processes. It is worth noting that the concept of “bridges” discussed here resembles that of “high betweenness” of previous studies on protein interaction networks using graph theory [26], [27]. If two clusters of interacting proteins are joined together only through a mutual interacting protein, this protein would have a “high betweeness” measure. “High betweeness” thus indicates the importance of a node within the wider context of the holistic network [27]. Here, the entropic contribution captures this property not in terms of shortest pats (as betweenness), but in terms of random walks inside the network. In this sense, network entropy and entropic contribution provide a conceptual framework to understand the role of the heuristic centrality indices, such as node degree and betweenness.

We are aware that our conclusion provides only one qualitative interpretation of the experimental observations. Under the assumption that gene dosage modifications in the ES cell lines represent small perturbations to the cellular system, more detailed theoretical interpretations can also be sought. For example, previous studies have described that cellular fates such as differentiation, growth, quiescence, or apoptosis may represent the convergence of stochastic cellular program onto a small set of common self-stabilizing “attractors” states [28]–[30]. These attractor states, which are robust to small perturbations, may also explain our observation that the transgenic ES cells remained in their original steady state as undifferentiated ES cells. However, we are cautious with respect to such a general conclusion, considering that our sample set is very limited, both in terms of sample dimension and its representative nature. Importantly, most of the current network data is of purely structural character, and does not allow for a more detailed understanding of the underlying dynamics, or even its logical abstraction. Moreover, the protein property information was obtained from current protein database entries that are incomplete and may be biased towards intensively studied proteins. Furthermore, due to our small sample sizes, the p-value estimations are not very robust, and may affect our assignment of significance for observed differences. Possible future experiments to test our hypothesis could be, for example, to analyze transgenic cell lines overexpressing one of the candidate balancer proteins in the same *in vitro* system.

In summary, based on our results we hypothesize that the large number of variant proteins detected in mutant ES cells does not necessarily reflect disease-related dysfunctions of these proteins, but rather a quantitative rearrangement of the proteome in response to a disturbance induced by gene dosage alterations. We postulated a regulatory mechanism established in a cell that protects it from deleterious effects of mutations by keeping the macromolecular composition of a cell quantitatively in balance.

## Materials and Methods

### Transgenic ES cell line construction

Pluripotent mouse ES cells were genetically manipulated on single or a set of genes involved in neurodegenerative diseases. A plasmid-mediated gene insertion protocol was used to generate *App* and *Snca*-overexpressing cell lines (*mES_hAPP* and *mES_Snca*, respectively), with CGR8 as parental line [31], [32]. For this purpose, a transfer vector based on pMSCV (BD BioSciences Clontech Heidelberg, Germany), which contained a puromycin resistance gene, was modified by inserting a 1.3 kb fragment of the rat promoter for translation elongation factor 1 alpha 1 (*Eef1a1*). This promoter has been shown to be suitable for protein overexpression in ES cells [33]. cDNA of a target gene (human *App* or mouse *Snca*) was inserted in frame with the initiating methionine specified by the rat *Eef1a1* promoter. The vector was electroporated into the ES cells at standard conditions (250V, 500 µF). 24h after electroporation, seven days of puromycin selection followed to select stably transformed ES cell lines. Western blotting was carried out to confirm protein overexpression (monoclonal mouse IgG against human amyloid β peptide, amino acids 1–17, clone 6E10; monoclonal mouse IgG against α-synuclein, clone 5D6, Signet Laboratories, Berkeley, USA). The MICER strategy was used to generate ES cell lines bearing segmental deletion or duplication of *Abcg1*-*U2af1* on mouse chromosome 17 (30333543 to 31387432 bp), using ES cell line HM-1 as parental line [13], [15], [34], [35] (*mES_14_Mono* and *mES_14_Tris*, respectively). This chromosome segment contains the following genes: *Abcg1*, *Tff3*, *Tff2*, *Tff1*, *Tmprss3*, *Ubash3a*, *Tsga2*, *LOC667056*, *Slc37a1*, *Pde9a*, *Wdr4*, *1500032D14Rik*, *Pknox1*, *Cbs* and *LOC623242*. The bordering gene *Abcg1* was deleted in the monosomy, but unaffected in the trisomy cell line. ES cell lines trisomic for murine *Dyrk1a* (*mES_Dyrk1a_Tris*) or murine *Dopey2* (*mES_Dopey2_Tris*) were generated using a BAC gene transfer protocol [36], with D3 as parental cell line (for *Dyrk1a:* BAC 189N10 from the CT7 library, pBeloBac11 vector, 94672437 to 94823558 bp on MMU16; for *Dopey2:* PAC 186P4 from the RP21 library, pPAC4 vector, 93576842 to 93751423 bp on MMU16) [37]. All ES cell lines were able to give germ-line transmission [14], [15], except for the CGR8 subclone used, which is primarily intended for work *in vitro* (Savatier, personal communication).

### Maintenance of ES cells

ES cell lines were grown in Dulbecco's Modified Eagle Medium (DMEM; Invitrogen, Karlsruhe, Germany) supplemented with 15% fetal calf serum (Biochrom, Berlin Germany), 2mM L-glutamine (Invitrogen), 0.1mM non-essential amino acids (Invitrogen), 1mM sodium pyruvate (Invitrogen), 0.1 mM 2-mercaptoethanol (Invitrogen) and 100U/ml leukemia inhibitory factor (LIF, Chemicon, Hampshire UK) under standard cell culture conditions (37°C, 5% CO2, 95% humidity). Modified and control cell lines were always cultured in parallel. CGR8-derived ES cell lines were maintained on gelatine-coated (0.1% v/v) cell culture plates. ES cells with E3 or HM-1 as parental line were maintained on mitotically inactivated murine embryonic fibroblasts. Prior to cell harvest, these cells were grown for three further passages on gelatine-coated plates to eliminate feeder cells. Cells were grown to 70–90% confluence and met morphological criteria for undifferentiated ES cells at the time of harvest (tightly packed cells forming round colonies). ES cells of three independent 10cm culture dishes were gently dissociated in ice cold PBS containing 5mM EDTA. This resulted in three biological replicates for each cell line. Trypsinization was avoided to preclude protein alteration artifacts.

### 2D-Electrophoresis

ES cell total protein extraction was carried out using our standard protocol [8]. 70 µg of protein was separated in each 2-DE-run as described previously [7]. Transgenic and their parental cell lines were always run in parallel. Two technical repeats were conducted for each cellular protein extract. Silver staining protocol was employed to visualize protein spots [38]. Computer-assisted manual gel evaluation was performed after scanning of the gel images (600 dpi, UMAX, Willich Germany) (Delta2D version 3.4, Decodon, Greifswald Germany) [39]. Briefly, corresponding gel images were first warped using “exact mode” (manual vector setting combined with automatic warping). A fusion gel image was subsequently generated using union mode, which is a weighted arithmetic mean across the entire gel series. Spot detection was carried out on this fusion image automatically, followed by manual spot editing. Subsequently, spots were transferred from fusion image to all gels. The signal intensities of each spot was computed as a weighted sum of all pixel intensities (volume of protein spot). Percent volume of spot intensities calculated as a fraction of the total spot volume of the parent gel was used for quantitative analysis of protein expression level. Ninety-five percent of the protein spots on the 2D gels that did not vary in their concentration and spot intensity served as reference. Thus, the balancing phenomenon is not due to a normalization artifact that could have arisen from global normalization to a mean or median. Normalized values after local background subtraction were subsequently exported from Delta2D in spreadsheet format for statistical analysis.

### Mass spectrometric protein identification

For protein identification by mass spectrometry, 2-DE gels were stained with a mass spectrometry compatible silver staining protocol [40]. Protein spots of interest were excised from 2-DE gels and subjected to in-gel trypsin digestion without reduction and alkylation. Tryptic fragments were analyzed on a LCQ Deca XP nano HPLC/ESI ion trap mass spectrometer (Thermo Fisher Scientific, Waltham, MA, USA) as described previously [11]. For database-assisted protein mass finger printing, monoisotopic mass values of peptides were searched against NCBI-nr (version 20061206, taxonomy: *Mus musculus*), allowing one missed cleavage. Peptide mass tolerance and fragment mass tolerance were set at 0.8 Dalton. Oxidation of methionine and arylamide adducts on cysteine (propionaide) were considered as variable peptide modifications. Criteria for positive identification of proteins were set according to the scoring algorithm delineated in Mascot (Matrix Science, London, UK) [41], with individual ion score cut-off threshold corresponding to p<0.05.

### Annotation of biochemical properties and functional categories to proteins

Public database queries were performed for the characterization of proteins with altered expression profiles in transgenic ES cells. For this purpose, *GOstat* (http://gostat.wehi.edu.au) was employed to annotate and search against the human GOA database (www.ebi.ac.uk/GOA) in order to determine highly represented functional categories for our proteins of interests [42]. This tool integrates a Fisher's exact test that decides whether the observed GO-term over-representation is significant. p<0.01 was set as statistical significance threshold. ProtParam was used to predict the protein instability, aliphatic index and Gravy scores (www.expasy.org/tools/protparam). The Human Reference Protein Database (www.hprd.org) was used to access the number of direct protein interaction partners. Batch searches of overall protein-protein interaction network information were performed via the meta-database UniHi (http://theoderich.fb3.mdc-berlin.de:8080/unihi). Subsequently, information originating from HPRD, BIND, DIP and Reactome, which are curated manually, was extracted. The visualization of the protein interaction network was performed using Cytoscape (www.cytoscape.org) [43]. The Mouse Genome informatics database (MGI 3.5) was used to access the number of SNPs across 86 inbred mouse strains (www.informatics.jax.org). Biological pathway analyses were performed using KEGG (www.genome.ad.jp/kegg) and Biocarta pathway databases (www.biocarta.com). Protein abundance information was extracted from 2-DE data.

### Statistics

To assess statistical significance of expression differences between transgenic and control cell lines, Student's T-test was carried out for control vs. transgenic groups (pair-wise, two sided, n = 6). p<0.05 was used as statistical significance threshold. Only protein expression changes over 30% compared to control were retained for further analysis. As a post hoc control analysis, protein expression data generated from 2DE were scrutinized using the Significance Analysis of Microarrays tool (SAM, www-stat.stanford.edu) to identify the false detective rate required to gain the comparable set of altered proteins (100 permutations) [44]. Protein expression alteration (fold change against wild-type controls) was reported with standard error of means (SEM). Due to their non-parametric distribution nature, protein property data (protein cellular abundance, biochemical properties and entropic contribution) were visualized as cumulative fraction plots. Differences of datasets between balancer and cell line-specific proteins were assessed with the Wilcoxon sum rank test (p<0.05).

### Network-based approach for system robustness analysis

Many aspects of cellular behavior are mainly determined by the structural properties of the underlying molecular network. In order to characterize the macroscopic resilience properties of the proteomic system, we adopted a network approach which is based on molecular protein interactions. This approach utilizes a fluctuation theorem [24], which states that the resilience of macroscoptic network observables is positively correlated with the pathway diversity, a property which can be measured by network entropy. In this context, network entropy appears as the dynamical entropy of a stochastic process defined on the network, i.e the weighted-average Shannon entropy, 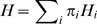, where π_i_ is the stationary distribution of the stochastic process (*P_ij_*) and *H_i_* is the standard Shannon entropy defined by:
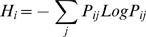
i.e., the uncertainty about the next step of a random walk operating on the network. The stochastic process, *P_ij_*, is defined through a variational principle for the leading eigenvalue, which, for unweighted networks, maximizes the overall network entropy [24]. Thus, “*H*” denotes the network entropy of the whole protein-protein interaction network, whereas “*H_i_*” denotes the entropic contribution of each individual protein (see Methods S1 for details). This entropic characterization leads to a natural importance ranking of proteins within the context of resilience of the global protein interaction network [25]. For this purpose, a protein-protein interaction network was generated from all proteins identified from the 2-DE protein pattern of ES cells. This generates an undirected, un-weighted information transfer graph where nodes denote proteins and the edges describe protein-protein interaction. The topological structure of the graph can be described by an NxN adjacency matrix A = (a_ij_). The entropic contribution of each protein to the global network entropy was calculated as in [25].

## Supporting Information

Table S1 Protein expression profile changes in transgenic ES cell lines.
(0.11 MB XLS)

Methods S1 Supplementary method of network entropy calculation
(0.05 MB PDF)
