# Supplementary material for: Proteomic Shifts in Embryonic Stem Cells with Gene Dose Modifications Suggest the Presence of Balancer Proteins in Protein Regulatory Networks
Source: PLoS One. 2007 Nov 28;2(11):e1218. doi: 10.1371/journal.pone.0001218 (PMC2077926; doi:10.1371/journal.pone.0001218)
Supplement: Methods S1 — Supplementary method of network entropy calculation (0.05 MB PDF) [file pone.0001218.s002.pdf]

Supplementary Material: Network Entropy

Here we briefly review the concept of network entropy and the fluctuation theorem, which had been introduced by Demetrius *et al.* [1], and applied to the characterisation of biological networks and their elements in [2].

The key ingredient in this formalism is a fluctation theorem which states that changes in the robustness of a network are positively correlated to changes in another macroscopic variable, network entropy. While robustness is defined as the resilience of the network against changes in the underlying network parameters, network entropy characterizes its pathway diversity. Importantly, network entropy can be specified in microscopic terms.

We start from a representation of the network in terms of its adjacency matrix, $A = (a_{ij})$, where the matrix elements are all non-negative to denote the interaction strength between nodes i and j in the network. Notice that for the special case of undirected and unweighted networks the adjacency matrix is Boolean and symmetric.

The largest (dominant) eigenvalue is a topological invariant of the adjacency matrix, and it is known to satisfy a variational principle [3]

$$\log \lambda = \sup_P \left[-\sum_{i,j} \pi_i p_{ij} \log p_{ij} + \sum_{i,j} \pi_i p_{ij} \log a_{ij} \right] \quad , \tag{1}$$

where the supremum is taken over all stochastic matrices $P = (p_{ij})$, that are compatible with the adjacency matrix A. Here compatible means that $p_{ij} = 0 \Leftrightarrow a_{ij} = 0$, and a stochastic matrix satisfies $\sum_j p_{ij} = 1$. The above formula also invokes the stationary distribution, π, which characterizes the long-time invariant behaviour of the Markov process described by the matrix P.

$$\pi P = \pi \quad . \tag{2}$$

If P is ergodic, then the components π_i satisfy $\pi_i > 0$ and denote the relative frequency with which the random walk on the network visits node i.

It has been shown [3] that, for strongly connected networks, the supremum is attained for a unique matrix $\hat{P} = (\hat{p}_{ij})$, where

$$\hat{p}_{ij} = \frac{a_{ij} v_j}{\lambda v_i} \quad . \tag{3}$$

With this choice of the matrix P, Eq. 1 becomes

$$\log \lambda = -\sum_{i,j} \pi_i \hat{p}_{ij} \log \hat{p}_{ij} + \sum_{i,j} \pi_i \hat{p}_{ij} \log a_{ij} \quad . \tag{4}$$

The first term on the right side is nothing but the network entropy,

$$H(\hat{P}) = -\sum_{i,j} \pi_i \hat{p}_{ij} \log \hat{p}_{ij} = \sum_i \pi_i H_i \quad , \tag{5}$$

where H_i is the standard Shannon entropy defined for each node i and π_i are the components of the stationary distribution as defined by Eq.2.

The fluctuation theorem derived in [1] introduces the probability $P_\epsilon(t)$ that time averages along trajectories differ by more than ϵ from ensemble averages over all trajectories. The ergodic theorem states that $P_\epsilon(t)$ converges to zero for large enough times. Hence one can define a fluctuation decay rate R as

$$R = \lim_{t\to\infty} \left[-\frac{1}{t} \log P_\epsilon(t) \right] \quad . \tag{6}$$

Large values of R entail small deviations of macroscopic observables from the ensemble average, and small values of R correspond to large fluctuations around its mean value. Thus, R characterizes the robustness of a macroscopic observable in the face of changes in the underlying parameters. The fluctuation theorem, [1], asserts that changes in R are positively correlated with changes in network entropy:

$$\Delta H \Delta R > 0 \quad . \tag{7}$$

The fluctuation theorem implies that an increase in entropy entails a greater insensitivity of macroscopic observables.

References

[1] L Demetrius, VM Gundlach, and G Ochs. Complexity and demographic stability in population models. *Theor Popul Biol*, 65(3):211–25, 2004.

[2] T Manke, L Demetrius, and M Vingron. An entropic characterization of protein interaction networks and cellular robustness. *J R Soc Interface*, 3(11):843–50, 2006.

[3] L Arnold, V Gundlach, and L Demetrius. Evolutionary formalism for products of positive random matrices. *Annals of Probab.*, 4(3):859–901, 1994.
